# Pre-diagnostic biomarkers of type 2 diabetes identified in the UAE’s obese national population using targeted metabolomics

**DOI:** 10.1038/s41598-020-73384-7

**Published:** 2020-10-19

**Authors:** Asma M. Fikri, Rosemary Smyth, Vijay Kumar, Zainab Al-Abadla, Salahedeen Abusnana, Michael R. Munday

**Affiliations:** 1 Ministry of Health and Prevention, Dubai, UAE; 2 Department of Pharmaceutical and Biological Chemistry, UCL School of Pharmacy, London, UK; 3 Rahsid Centre for Diabetes and Research, Ajman, UAE

**Keywords:** Biomarkers, Predictive markers, Biochemistry, Metabolomics, Endocrinology, Endocrine system and metabolic diseases, Diabetes, Metabolic syndrome, Obesity, Pre-diabetes, Mechanisms of disease

## Abstract

Currently, type 2 diabetes mellitus (T2DM) and obesity are major global public health issues, and their prevalence in the United Arab Emirates (UAE) are among the highest in the world. In 2019, The UAE diabetes national prevalence was 15.4%. In recent years there has been a considerable investigation of predictive biomarkers associated with these conditions. This study analysed fasting (8 h) blood samples from an obese, normoglycemic cohort and an obese, T2DM cohort of UAE nationals, employing clinical chemistry analysis, 1D ^1^H NMR and mass spectroscopy (FIA-MS/MS and LC-MS/MS) techniques. The novel findings reported for the first time in a UAE population revealed significant differences in a number of metabolites in the T2DM cohort. Metabolic fingerprints identified by NMR included BCAAs, trimethylamine N-oxide, β-hydroxybutyrate, trimethyl uric acid, and alanine. A targeted MS approach showed significant differences in lysophosphatidylcholines, phosphatidylcholines, acylcarnitine, amino acids and sphingomyelins; Lyso.PC.a.C18.0, PC.ae.C34.2, C3.DC..C4.OH, glutamine and SM.C16.1, being the most significant metabolites. Pearson’s correlation studies showed associations between these metabolites and the clinical chemistry parameters across both cohorts. This report identified differences in metabolites in response to T2DM in agreement with many published population studies. This contributes to the global search for a bank of metabolite biomarkers that can predict the advent of T2DM and give insight to its pathogenic mechanisms.

## Introduction

Non-communicable diseases (NCDs) are a major health problem in the United Arab Emirates (UAE) and were the leading cause of mortality in UAE, accounting for 77% of total deaths in 2016 of which 5% were due to diabetes^1^. In 2009 there were 285 million people globally with diabetes but by 2019 this had increased to 463 million and represented 9.3% of the global adult population aged 20–79 years^2^. This rate of increase was evident in the UAE where rapid economic growth and prosperity produced lifestyle changes that paralleled a rise in diabetes and its clinical complications. In 2017, in the UAE, 1,185,500 adults were diagnosed with diabetes which accounted for 17.3% of the general population^3^. However, UAE nationals represent only about 20% of the total population and Hamoudi and co-workers reported in 2019 that a higher proportion of UAE nationals (21% of male and 23% of female) are diabetic^4^. The majority of diabetic patients suffer from Type 2 Diabetes Mellitus (T2DM) that develops in middle age and a worrying global statistic is the number of pre-diabetic patients who are likely to progress to full blown T2DM. The prevalence of pre-diabetes varies between countries but globally (7.3% in 2017) it is thought to affect almost the same percentage of population that suffer T2DM^5^. In the UAE, in addition to the 21–23% of UAE nationals that were diabetic, a further 20% were reported to be pre-diabetic^4^. Globally, pre-diabetes^5^ and T2DM^2^ are increasing and this is ascribed to the increased prevalence of behavioural and metabolic risk factors such as hyperglycaemia, hyperlipidaemia, hypertension, obesity, reduced physical inactivity and smoking^1^. T2DM causes many long-term complications associated with increased rates of mortality and morbidity. The most important complications in this context are heart disease, stroke, kidney failure (nephropathy), peripheral nerve damage and peripheral vascular disease^6^. In 2017, diabetes accounted for 10.7% of deaths in 20–79 years olds globally, and healthcare expenditure of USD 727 billion^3^. The International Diabetes Federation (IDF) reported in 2019 that the mean diabetes related expenditure per person with diabetes in the UAE is 1,237.3 USD^2^. This represents a serious economic and health burden that many countries, including the UAE, are attempting to alleviate through investment in research.

Early and accurate diagnosis of pre-diabetes has been identified as an important goal in the UAE population^7^. This project was carried out as one of many contributions to national diabetes research in the UAE.

There are a number of recognized biomarkers for T2DM risk, such as fasting plasma glucose and glycated hemoglobin A_1c_ (HbA_1c_)^8^, triglycerides^9^, adiponectin and inflammatory markers^10^. However, most of these do not provide sufficient early predictive power for insulin resistance and prediabetes. Consequently, there have been considerable advances in recent years to identify changes in the serum metabolome of obese, and prediabetic and T2DM patients^11,12^. This has been in the hope of identifying specific metabolite biomarkers that more rapidly reflect the physiological changes in the early stages of the disease. While no single metabolite truly fulfils the role of biomarker, it is clear that changes in a number of blood metabolites accompany the various stages of the development of T2DM and may have some predictive power for this process. Branched chain amino acids (BCAA) such as valine, leucine and isoleucine, along with phospholipids were among the most popular biomarkers associated with T2DM progression^11,12^.

Wang and co-workers demonstrated increases in the plasma branched chain amino acids (BCAA) leucine, isoleucine and valine, and the aromatic amino acids phenylalanine and tyrosine, in patients that developed T2DM over a 12-year period compared to age-, sex- and BMI-matched controls from the same cohort^13^. Increased plasma levels of BCAA have since been confirmed in patients with increased free fat mass index in the population-based Cooperative Health Research study in the Ausburg region (KORA)^14^ and in cohorts of women in the UK who were T2DM or had impaired fasting glucose^15^.

A recent study by Vogelzangs et al*.*^16^ in European patients showed that hepatic and muscular insulin resistance was associated with significantly higher plasma levels of valine, isoleucine, oxo-isovaleric acid, alanine, lactate, and triglycerides, and lower levels of glycine^16^.

Wang-Sattler et al.^17^ quantified 140 metabolites for 4,297 fasting patient serum samples using a cross-sectional approach in the KORA study and found that glycine, lysophosphatidylcholine (LPC) and acetyl carnitine levels were significantly altered in individuals with impaired glucose tolerance (IGT) compared to those with normal glucose tolerance^17^. The lower levels of glycine and LPC were found to predict not only IGT but also T2DM even 7 years before disease onset. Using metabolite-protein network analysis, the group found that T2DM-linked genes PPARG, TCF7L2, HNF1A, GCK, IGF1, IRS1 and IDE were linked to these metabolite changes^17^. The changes in plasma glycine, LPC and acyl carnitine where also found to be associated with T2DM in a Korean population^18^. This study also identified phosphatidylcholine acyl alkyl 36:0 as a significant indicator of T2DM in this population and linked these alterations in metabolites to ten genetic variable loci some of which had been previously implicated in T2DM or obesity^18^. Analysis of serum from patients in the German EPIC-Potsdam clinical study also revealed an increase in diacyl phosphatidylcholines C32:1, C36:1, C38:3 and C40:5 to be independently associated with an increased risk of T2DM^19^. A recent study examined the association of pairwise metabolite ratios with insulin secretion in patients from the Netherlands. It demonstrated that the valine to phosphatidylcholine acyl-alkyl C32:2 ratio showed positive association with oral glucose tolerance measured insulin secretion and insulin resistance^20^.

Most of these studies used cross-sectional or prospective methods to investigate metabolites related to T2DM in German^14,17,19^, UK^15^, USA^13^ Dutch^20^, pan-European^16^ and even Korean^18^ populations. However, studies in Middle Eastern and Arabic populations are rare. Even more so for the UAE national population despite its propensity for T2DM. In this project, for the first time, the metabolite profiles of local Emirati individuals with T2DM have been examined.

### Aim

The aim of this study was to use untargeted and targeted metabolomic techniques to examine the metabolite profiles of obese T2DM Emirati patients from the UAE, in comparison to obese controls who did not exhibit T2DM. This would allow the identification of differences in individual metabolites that might be indicative of the T2DM condition in this population. This data would contribute to a global comparison of metabolomic changes in different populations in response to insulin resistance, prediabetes and T2DM as researchers hope to identify a bank of metabolite biomarkers that might be potential predictors of these conditions.

## Results

### Physical and clinical parameters

The physical parameters indicate that all patients were obese as defined by a BMI ratio greater than 30 which is globally recognised as an indication of obesity^21^. Clinical chemistry analysis showed significant increases in the fasting blood glucose (67.9%) and HbA1c (49.5%) in the obese T2DM group compared to obese controls (Table 1). Total cholesterol and LDL levels were high or borderline high in all groups but were significantly higher in the obese controls than in the obese T2DM group. HDL levels were within the normal range in all groups except the obese men (Table 1).

**Table 1:** Physical and clinical chemistry measurements (expressed as the mean ± SD) of obese men and women. All patients were male/female UAE nationals.

	Reference range			Unit	Obese individuals (n = 50)		Obese T2DM patients (n = 50)	
	Sex	Low	High		Men (n = 20)	Women (n = 30)	Men (n = 24)	Women (n = 26)
Age at recruitment				Years	32.1 ± 9.2	39.0 ± 8.9	46.4 ± 10.2	48.7 ± 11.0
Body weight				kg	113.1 ± 25.7	91.4 ± 14.6	102.2 ± 13.6	95.5 ± 20.2
Height				cm	173.6 ± 11.9	157.4 ± 6.4	170.5 ± 6.6	157.2 ± 7.0
Waist circumference				cm	113.3 ± 12.8	99.1 ± 9.5	112.9 ± 9.2	112.3 ± 15.1
BMI	M/F			kg/m^2^	37.3 ± 6.1	36.8 ± 4.8	35.2 ± 4.7	38.7 ± 7.9
Systolic blood pressure	M/F	< 100	> 120	mmHg	134.5 ± 12.8	128.3 ± 11.9	141.1 ± 16.5	136.3 ± 18.2
Diastolic blood pressure	M/F	< 60	> 80	mmHg	83.5 ± 8.7	73.3 ± 10.2	82 ± 9.4	75 ± 11.3
Heart rate	M/F	< 60	> 100	bpm	82.9 ± 10.8	80.4 ± 9.5	79.7 ± 10.9	82.7 ± 9.3
Glucose (fasting)***	M/F	4.11	6.05	mmol/L	5.8 ± 0.7	5.4 ± 0.5	9.6 ± 4.0	9.2 ± 3.2
HbA1c***	M/F	4.8	5.9	%	5.2 ± 0.5	5.1 ± 0.3	7.9 ± 1.7	7.4 ± 1.4
Insulin	M/F	2.6	24.9	µU/ml	31.2 ± 9.9	17.0 ± 6.8	16.9 ± 9.4	17.7 ± 9.5
C peptide	M/F	0.37	1.47	nmol/L	1.4 ± 0.7	1.0 ± 0.2	1.1 ± 0.5	0.9 ± 0.3
Cardiac C-reactive protein	M/F	0	5	mg /L	4.2 ± 2.8	5.7 ± 4.0	6.9 ± 14.5	11.3 ± 19.0
Total cholesterol**	M/F	0	5.2	mmol/L	4.9 ± 1.0	4.8 ± 1.2	4.0 ± 1.0	4.2 ± 0.8
Triglycerides	M/F	0	2.26	mmol/L	2.8 ± 4.0	1.2 ± 0.5	1.8 ± 0.9	1.5 ± 0.9
High density lipoprotein (HDL)	M	< 1.0	> 1.3	mmol/L	0.9 ± 0.3	1.4 ± 0.3	1.02 ± 0.3	1.3 ± 0.4
	F	< 1.3	> 1.5					
Low density lipoprotein (LDL)**	M/F	0	2.59	mmol/L	3.35 ± 0.9	3.2 ± 1.3	2.4 ± 0.9	2.7 ± 0.9

Patients were stratified into obese non-diabetic and obese T2DM groups on the basis of fasting blood glucose and HbAlc levels. Samples of the two study groups (n = 50 each) were compared using Student’s t-test. Significance levels expressed as*P < 0.05; **P < 0.01; ***P < 0.001.

### 1D ^1^H NMR metabolomics analysis

**Figure 1:**
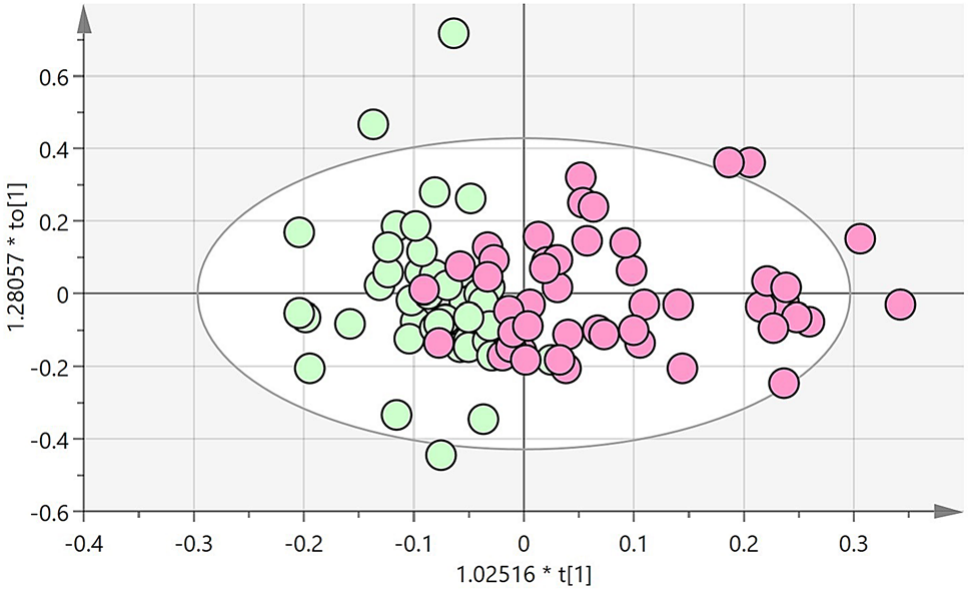
Scores plot from OPLS-DA model derived from 1D ^1^H NMR spectral data of 8 h fasted plasma samples from obese control (n = 50) and obese T2DM (n = 50) subjects. NMR preparation, data processing and integration was carried out as described in the methods section. Every spot in the plot represents one plasma sample. Green: obese control samples; Pink: obese T2DM patient samples.

1D ^1^H NMR metabolomic analysis revealed significant changes in chemical shifts between the obese control and obese T2DM groups with male and female samples combined. Figure 1 shows that in an OPLS-DA score plot of plasma samples from the two groups, obese control samples are negative for discriminant component t[1] whereas obese T2DM samples are positive for t[1]. This separation of metabolite profiles was also seen when obese control males were compared to obese T2DM males and when obese control females were compared to obese T2DM females (data not shown). The spots in Fig. 1 were then compared with their corresponding individual clinical chemistry data. Samples overlapping in the middle region belonged to control individuals who had blood profiles with fasting blood glucose and HbA1c at the higher end of the distribution in the group and T2DM individuals with lower values within the range of distribution in that group. The spots at the extremities of each distribution in Fig. 1 were samples of control individuals with normal plasma glucose and HbA1c profiles or T2DM individuals with higher plasma levels of glucose and HbA1c.

The OPLS-DA model identified chemical shifts with VIP values > 1 and these were tentatively identified by reference to databases as described in the Methods section. A list of possible metabolites whose concentration are either increased or decreased significantly (*P* < 0.05) in the obese T2DM patients compared to obese controls, are presented in Table 2. In the T2DM patients there were significant increases in the plasma concentration of the BCAAs leucine and isoleucine, trimethylamine-N-oxide (TMAO), thymidine, glycerol, trimethyl uric acid, β-OH butyrate and, not surprisingly, glucose. Slightly less significant were increases in taurine, malonate and uric acid (Table 2). Alanine was very significantly decreased in the plasma of T2DM patients with less significant decreases in acetate, lactate, hexanoyl carnitine, uridine, and in keeping with the clinical chemistry data, cholesterol (Table 2).

**Table 2:** OPLS-DA-derived VIP-plot detected chemical shifts responsible for the separation of 1D ^1^H NMR spectral data of 8 h fasted plasma samples from obese controls (n = 50) and obese T2DM (n = 50) patients.

Metabolite name	Chemical shift (δ) and type	*P*-value (Increased ↑) or (decreased ↓)
β-hydroxybutyrate	1.18 (d)	0.009 (↑)
Acetate	1.9 (s)	0.042 (↓)
TMAO	3.26 (s)	2.61E-06 (↑)
Taurine	3.4 (t)	0.004 (↑)
d-Glucose	3.46 (m), 3.38 (m), 3.62 (d), 3.82 (m), 3.72 (m)	7.49E-12 (↑)
Leucine	1.7 (m), 3.7 (m)	0.003 (↑)
Isoleucine	3.66 (d), 0.98 (d)	4.36E−10 (↑)
1, 3, 7-Trimethyl uric acid	3.34 (s), 3.39 (s)	0.002 (↑)
Glycerol	3.58 (dd)	6.27E−07 (↑)
TMAO	3.26 (s)	2.61E−06 (↑)
Thymidine	3.7 (m), 4.01 (q)	0.004 (↑)
Alanine	1.46 (d), 3.75 (q)	7.98E−12 (↓)
Cholesterol	0.86 (dd), 0.90 (d), 1.82 (d), 1.32(s), 1.5 (s)	0.017 (↓)
Malonate	3.1 (s)	0.019 (↑)
α-Lactate	4.1 (m), 1.3 (m)	0.026 (↓)
Hexanoylcarnitine	0.86 (t)	0.037 (↓)
Uridine	4.34 (dd)	0.018 (↓)
Cholesterol sulfate	0.66 (m), 0.94 (m), 1.78 (m)	0.036 (↓)
Uric acid	3.1 (t)	0.031 (↑)

Chemical shift order based on VIP score larger than 1; from highest value to lowest. Plasma samples were collected, and metabolites were extracted for NMR analysis as described in methods. Chemical shifts were statistically compared by means of a student’s t-test. (s) Singlet, (d) Doublet, (dd) Double Doublet, (t) Triplet, (m) Multiple, (q) Quadruplet. Values that differ significantly from controls are shown: *P < 0.05; **P < 0.01; ***P < 0.001. (↑) Increased, (↓) Decreased; in obese T2DM samples compared to obese controls.

### Targeted metabolomics (FIA-MS/MS and LC-MS/MS)

Targeted metabolomics employing the Biocrates AbsoluteIDQ p180 Kit assay using FIA-MS/MS and LC-MS/MS identified and quantified 143 metabolites of which 42 were significantly different between the obese control and obese T2DM groups. The 25 metabolites that had strong power and effect size are shown in Table 3. The other 118 metabolites (including their mean concentration, *P*-value and q-value) can be found online as supplementary, Table S1.

**Table 3:** Targeted mass spectroscopy quantified significantly altered fasting plasma metabolites with *P* and q values < 0.05 in obese controls (n = 40) and obese + T2DM patients (n = 40).

Metabolite class	Metabolite	Control mean conc	T2DM mean conc	*P*-value	q value (BH adjusted)	Effect size	Power (%)
Sugars	H1	4989.75	7399.65	1.40E−09	2.00E−07	1.336	100.0
Phosphatidylcholines	PC.ae.C34.2	8.1888	5.8055	2.75E−08	1.97E−06	1.389	100.0
	PC.ae.C36.3	5.7717	4.2237	2.90E−07	1.38E−05	1.246	99.9
	PC.ae.C36.4	14.6275	11.5653	3.97E−07	1.42E−05	1.207	99.9
	PC.aa.C36.2	171.5	138.66	2.19E−06	5.23E−05	1.065	99.9
	PC.aa.C32.3	0.4256	0.3185	3.34E−06	6.81E−05	1.043	99.9
	PC.ae.C38.5	11.0617	9.3667	7.15E−05	0.0009	0.867	99.0
	PC.aa.C34.2	272.85	234.9	8.85E−05	0.0011	0.863	99.0
	PC.ae.C34.3	5.8365	4.4625	0.0002	0.0018	0.834	98.6
	PC.aa.C36.3	107.5975	91.1925	0.0002	0.0019	0.840	98.6
	PC.aa.C38.3	38.03	31.3475	0.0005	0.0040	0.778	97.1
Lysophosphatidylcholines	lysoPC.a.C18.0	20.8625	17.3475	0.0007	0.0055	0.810	98.0
	lysoPC.a.C18.2	16.5508	12.7825	0.0021	0.0111	0.749	96.0
	lysoPC.a.C18.1	0.2607	0.2068	0.0128	0.0446	0.631	87.8
Sphingomyelins	SM.C16.1	20.225	16.67	5.42E−05	0.0008	0.925	99.6
	SM..OH..C14.1	6.873	5.5665	0.0002	0.0021	0.875	99.1
	SM.C20.2	0.491	0.3835	0.0004	0.0037	0.763	96.6
	SM..OH..C22.2	10.9228	9.164	0.0016	0.0091	0.735	95.4
	SM..OH..C16.1	3.368	2.882	0.0141	0.048	0.575	81.3
Acylcarnitines	C3 DC..C4.OH	0.0395	0.0607	9.28E−07	2.66E−05	0.988	99.8
Amino acids	Gln	560.5	480.175	0.0002	0.0018	0.890	99.3
	His	87.66	77.7075	0.001	0.0066	0.722	94.7
	Ala	315.775	379.6	0.001	0.0066	0.760	96.4
	Orn	62.045	50.2025	0.0011	0.0074	0.715	94.3
	Trp	66.92	58.235	0.0013	0.0082	0.711	94.1

All patients were obese male/female UAE nationals. Subjects were stratified into obese non-diabetic and obese T2DM groups. The mean plasma concentration (µM) of metabolites with *P* and q values < 0.05 and power ≥ 80% was reported. Statistical significance (*P*) was calculated using Student’s t-test, and the results were adjusted for q value (Benjamini–Hochberg adjustment). Post hoc: difference between independent means analyses was applied to calculate effect size and power.

Not surprisingly, total hexose (H1) was significantly higher in the T2DM group (*P* = 1.4E−09, q = 2.0E−07). However, other differences in plasma metabolites were found to be significantly decreased levels of phosphatidylcholines, LPCs, sphingomyelins, glutamine, histidine, ornithine and tryptophan in the T2DM group. Whereas acyl carnitines and alanine were significantly increased in the T2DM group (Table 3).

**Figure 2:**
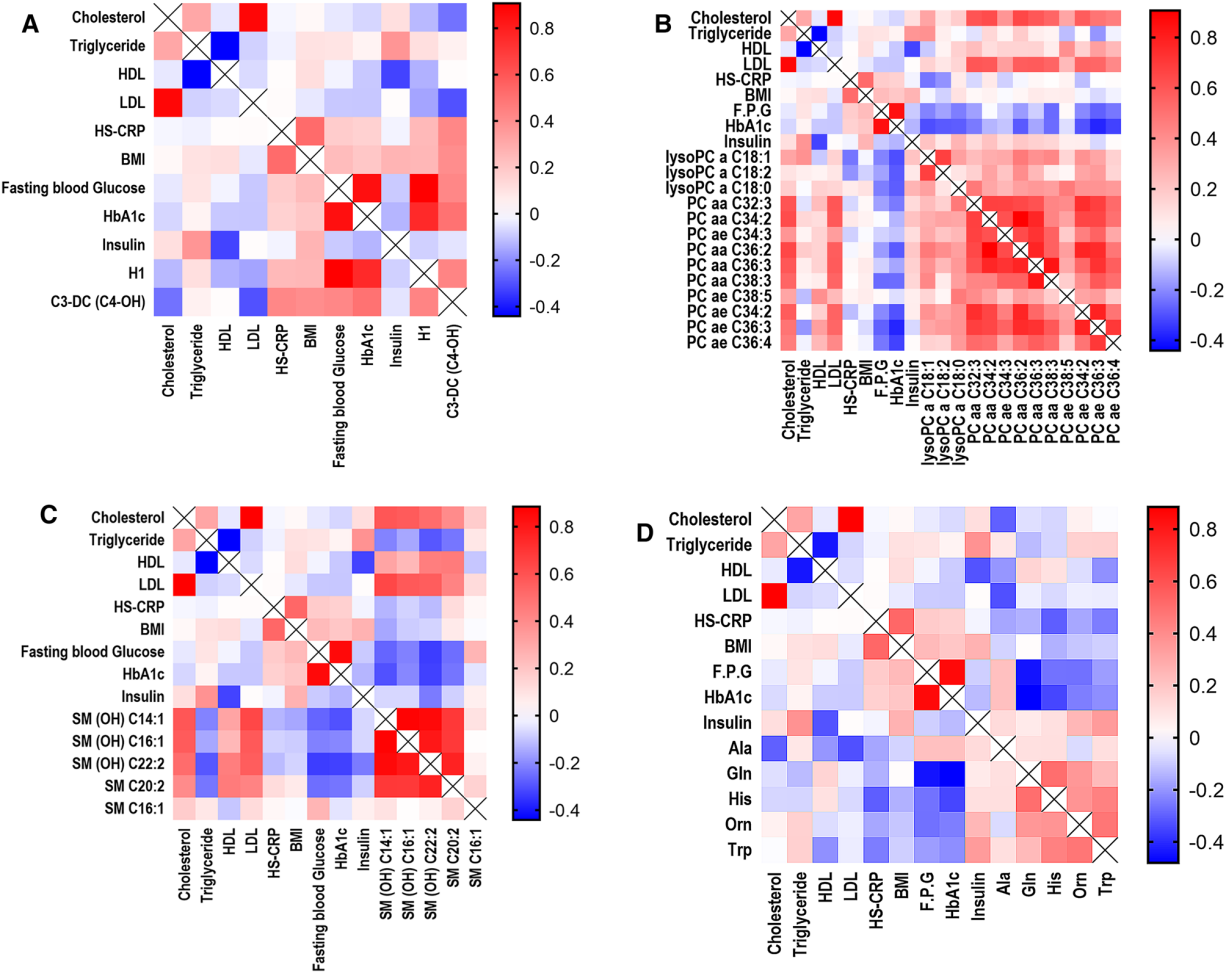
Pearson’s correlation coefficient (r) of obese controls and obese + T2DM patients’ fasting plasma measurements of clinical chemistry biomarkers with (**a**) hexose (H1) and acylcarnitine C3-DC (C4-OH); (**b**) phosphatidylcholines and lysophosphatidylcholines; (**c**) sphingomyelins; (**d**) amino acids and biogenic amines, quantified using Biocrates AbsoluteIDQ p180. All subjects were male/female UAE nationals. The mean plasma concentration (µM) was compared. Each coloured square corresponds with the (r) value for the correlated parameters, and the ligand rainbow colour theme corresponds to the numeric (r) value. (r) value ranges from − 1 to 1; a value of zero means no correlation at all. Fasting plasma glucose (FPG). The metabolites shown are of highly significant *P*- and q values selected based on Table 2.

In the current study, the correlation between the clinical chemistry biomarkers and metabolomics-identified markers was investigated using Pearson’s correlation analyses. The clinical chemistry parameters added as covariates with the significantly altered metabolite classes identified by targeted MS/MS include cholesterol, triglyceride, LDL, HDL, HS-CRP, BMI, fasting plasma glucose (FPG), insulin, and HbA1c (Fig. 2). Phosphatidylcholines, acylcarnitines, sphingomyelins, amino acids and biogenic amines, and LPCs were strongly correlated with the clinical biomarkers, as shown in Fig. 2.

## Discussion

In this study, physical and clinical chemistry parameters were used to stratify the UAE patients into two groups. Consequently, while all patients were obese with a BMI in excess of 30, the T2DM were distinguished on the basis of high fasting blood glucose and high HbA1c (Table 1). However, there were other differences in that total cholesterol levels and LDL were significantly higher in the obese control group than those in the obese T2DM group. This may reflect positive effects of antidiabetic therapy on circulating lipid levels in the T2DM group, although there were no significant differences in triglyceride levels (Table 1).

Untargeted 1D ^1^H NMR metabolomic analysis revealed a clear separation between the plasma metabolomes of the two groups as demonstrated by the OPLS-DA model shown in Fig. 1. While increased plasma glucose in the T2DM group will have contributed to the clustering differences found in the model, it was clear that there were a number of other contributory chemical shifts (Table 2). The significant increase in BCAAs in the obese T2DM group compared to obese controls is in agreement with previous findings that report increases of BCAAs associated with T2DM^13–16^. Both leucine and isoleucine were found to be increased in the MS data, however, the difference was not significant and thus not included in Table 3. Decreased expression of the enzymes involved in BCAA catabolism has been reported as a mechanism that explains their increased plasma concentration in T2DM^22^. The BCAA in turn have been implicated in the activation of mammalian target of rapamycin complex 1 (mTORC1) to cause insulin resistance^23^. The significant increase in trimethylamine N-oxide (TMAO) may reflect an emerging relationship with carnitine levels that is reported to change in T2DM. Genetically diabetic (db/db) mice were shown to have tenfold higher plasma levels of TMAO and lower L-carnitine levels compared to non-diabetic controls^24^ and in the same study both pre-diabetic and diabetic patients showed higher plasma TMAO levels than non-diabetic^24^.

Less significant changes in glycerol, β-OH butyrate, malonate, lactate, acetate etc. were observed and reflect changes in energy utilization, glucose metabolism and uptake and other perturbations of the underlying conditions as reported previously for insulin resistant patients^11,12,25^. Of note is the highly significant decrease in plasma alanine in the obese T2DM group in this study (Table 2) which is in agreement with a decrease in a range of amino acids, including alanine, observed in a cohort of Italian T2DM patients^26^.

NMR analysis provided good evidence for an altered pattern of metabolites between the groups and confirmation that some interesting metabolites recognized in other populations are also changing in response to T2DM in UAE nationals. Nonetheless, using one approach alone can be limiting. For example, spectral crowding and overlap results in difficulties assigning spectra to metabolites. Thus, another platform was used for metabolomic analysis in the form of FIA-MS/MS and LC-MS/MS. Unsurprisingly, total hexose (H1), which is mainly represented by glucose, was one of the metabolites detected by mass spectrometry and altered in the T2DM group with the greatest effect size and power (*P* = 1.4E−09, q = 2.0E−07, Table 3). However, there were many other metabolites with almost equally powerful levels of change.

Phosphatidylcholines were ranked second in terms of effect size and power as a class of compounds whose plasma levels were lower in the obese T2DM group compared to obese controls (Table 3). Diacyl-phosphatidylcholines consist of glycerol linked to phosphocholine and two fatty acid residues, and removal of one fatty acid produces LPCs. Phospholipids are the main constituents of cellular membranes and are subsequently involved in cellular signal transduction^27^. Diacyl-phosphatidylcholines (PCn aa Cn) are crucial for the secretion of VLDL and HDL from hepatocytes. On the other hand, acyl-alkyl-phosphatidylcholines (PCn ae Cn) possibly act as antioxidants preventing lipoprotein oxidation^27^. In this study, ten species of phosphatidylcholine (PC ae C34.2, PC ae C36.3, PC ae C36.4, PC aa C36.2, PC aa C32.3, PC ae C38.5, PC aa C34.0.2, PC ae C34.3, PC aa C36.3, and PC aa C38.3) were significantly decreased by 15–30% in the obese T2DM group compared to the obese controls (Table 3). Several publications report changes in plasma phosphatidylcholine concentration associated with T2DM^18–20^, or a shift from acyl-alky phosphatidylcholine to acyl-acyl phosphatidylcholines^14^. Reduced plasma levels of phosphatidyl choline and phosphatidyl ethanolamine were observed in patients with impaired fasting glycaemia or T2DM^28^. Significant reductions in plasma concentrations of linoleoyl-glycerophosphocholine have been reported in patients with insulin resistance (18%) or T2DM (55%)^29^.

Lysophosphatidylcholines (LPCs) are a subgroup of the glycerophospholipid family and lyso.PC.a.C18.0, lyso.PC.a.C18.2 and lyso.PC.a.C28.0 were significantly decreased approximately 20% in obese T2DM patients compared to obese controls (Table 3). This is in agreement with many previous studies that report a decrease in LPC in other populations of T2DM patients^17–19,28^. LPCs have many functions, such as carriers for essential fatty acids; influencing hepatic metabolism by inhibiting hepatic cholesterol biosynthesis; activation of PPAR-α and -β; inhibition of insulin-dependent glycogen synthesis and β-oxidation^27,30^.

Klingler et al. suggested that LPCs might be of clinical interest, not only as biomarkers but also as activators of PPAR-δ that would protect skeletal muscle from lipotoxicity^30^.

Plasma acylcarnitines were significantly higher by 54% in the T2DM group than in the controls (*P* = 9.2840E−07; q = 2.6552E−05, Table 3). Acetyl carnitine is produced in the mitochondrial matrix by the enzyme, carnitine-O-acetyl transferase (CrAT), from carnitine and acetyl-CoA. It is the most abundant form of the carnitines that are involved in fatty acid transport into mitochondria and are crucial for maintaining normal mitochondrial function^31^. Lipid oversupply is recognised as a cause of insulin resistance and so carnitine is essential to the reduction of the toxic effects of fatty acyl-CoA^32^. Several previous studies have reported an increase in plasma acyl carnitines in patients with T2DM^14,17,18^ and this increase has been attributed to incomplete long chain fatty acyl-CoA oxidation^33^.

The plasma sphingomyelins were significantly decreased by 15–22% in the T2DM obese group compared to controls (Table 3). Sphingomyelin plays a role in cell apoptosis by hydrolysing into ceramide that is in turn responsible for the induction of cell differentiation, inhibition of cell proliferation, induction of apoptosis and involvement in inflammatory processes. Ceramide is involved in insulin-mediated glucose uptake, inhibition of insulin-stimulated glucose uptake and translocation of GLUT1 and GLUT4 to the plasma membrane in 3T3-L1 adipocytes^34^ and is thought to play an essential role in the development of insulin resistance^35^. The findings in this study are in agreement with reduced plasma levels of sphingomyelins that have been reported in other T2DM patients^19,28^.

Mass spectrometric analysis revealed five amino acids and biogenic amines that were significantly changed in the obese T2DM patients compared to obese controls (Table 3). There was a decrease in glutamine (14%), histidine (11%), ornithine (19%) and tryptophan (13%), and an increase (20%) in alanine (Table 3). Changes in amino acids and intermediates of the urea cycle have been widely reported to change in the plasma of insulin resistance or T2DM patients^11,12^.

In the current study, the correlation between the clinical chemistry biomarkers and the targeted metabolomics-identified metabolites for all patients was investigated using Pearson’s correlation analyses. The clinical chemistry parameters were added as covariates with the significantly altered metabolite classes identified by targeted MS/MS (Fig. 2). Figure 2a, shows unsurprisingly that hexose (H1) is very strongly positively correlated with Fasting Plasma Glucose (FPG) and HbA1c and cholesterol very strongly positively correlated with LDL. However, acylcarnitine C3-DC (C4-OH) shows a positive correlation with HbA1c, FPG, H1 hexose, BMI and the inflammatory marker HS-CRP. This is consistent with the observation that plasma acylcarnitines are elevated in T2DM and reflect an inability to cope with fatty acyl-CoA promotion of insulin resistance^32^. The positive correlation between acylcarnitine and HS-CRP supports the suggested relationship between inflammation and mitochondrial dysfunction in this process. It is supported by a previous observation that acylcarnitines activate proinflammatory pathways^33^. More difficult to explain is an inverse correlation between acylcarnitine C3-DC (C4-OH) and LDL and cholesterol (Fig. 2a).

Figure 2b shows a strong positive correlation between the phosphatidylcholines, cholesterol and LDL. However, an inverse correlation with HbA1c and FPG depends very much on the species of phosphatidylcholine. PC ae C36:3 and PC ae C36:4 show much stronger inverse correlations with HbA1c and FPG than PC ae C38:5 and PC ae C34:3 that show no correlation at all. This suggests that individual phosphatidylcholines may vary with respect to their predictive capabilities in T2DM. Figure 2b reveals that LPCs are strongly inversely correlated with FPG and HbA1c but have no correlation with cholesterol or LDL.

Figure 2c shows a strong positive correlation between sphingomyelins and cholesterol, HDL and LDL and a strong inverse correlation between sphingomyelins and FPG, HbA1c and triglycerides. This inverse correlation is supported by published literature^19,28^.

Figure 2d shows variable relationships with a tendency to inverse correlation of histidine, ornithine and tryptophan with FPG and HbA1c. However, there was a very strong inverse correlation between glutamine and FPG and HbA1c (Fig. 2d). High glutamate: low glutamine ratios have previously been reported in Spanish T2DM patients^36^.

It is important to highlight the possibility of medication effects on the T2DM group metabolite profiles which might present a limitation to the study. Not many metabolomics studies are published addressing these effects comprehensively. Two studies on metformin showed significant changes in PC ae C36:4, PC ae C38:5, PC ae C38:6 and citrulline due to its use. Although these metabolites are reported in this study and might be affected by metformin use, there is no published evidence about its effects on the other metabolites^37,38^.

In summary, T2DM is a multifactorial condition that involves dysregulation of signal transduction, cellular homeostasis, cell apoptosis, dysfunctional adipose, and chronic low-grade inflammation, among others. Early detection of these dysfunctions through biomarkers such as changes in metabolite patterns will allow treatment and prevention at the stage of prediabetes before full blown T2DM. This explains the expansion of research into T2DM metabolomics in recent years and the identification of metabolites associated with obesity, insulin resistance, prediabetes and T2DM in populations across the world. The current study has analysed a limited population of UAE nationals that were all classified as obese but who were stratified into a normoglycemic, non-diabetic cohort or one exhibiting T2DM. It has for the first time identified and confirmed an interesting pattern of differences in metabolites profiles in T2DM patients from the UAE that have in many cases also been identified in various other international patient cohorts. This is a useful contribution to the global quest for a bank of metabolite biomarkers that can predict the advent of T2DM and give insight to its pathogenic mechanisms. Further investigations of patient cohorts in the UAE where T2DM is so prevalent are clearly warranted.

## Methods

### Patients

This collaborative cross-sectional study was carried out at the Rashid Centre for Diabetes and Research, Ajman—UAE. Patients (N = 100) were male/female UAE nationals aged between 18 and 60 year, with a BMI ≥ 30 kg/m^2^ (obese) and no serious medical conditions/major surgeries. Patients were stratified into obese non-diabetic (n = 50) and obese T2DM (n = 50) groups based on fasting blood glucose and HbAlc levels and diagnosis confirmed by the clinician in charge (Table 1). T2DM patients were receiving one or multiple of the following medications: Metformin, Sitagliptin, Insulin, Atorvastatin, Simvastatin and Aspirin (prophylaxis).

All candidates were interviewed and asked to give informed consent. Informed consent was obtained from all candidates participating in this study. All procedures were carried out following the guidelines of good clinical practice and all experimental protocols were approved by the concerned institutional and/or licensing committees; the ethics committees of University College London (UCL) and the UAE Ministry of Health and Prevention (UCL Ethics Application No 7457/001 and UAE Ministry of Health and Prevention Ethics Application No MOHP/REC/12). The data collected during the project were coded and anonymised for patient confidentiality purposes.

### Sample collection and clinical chemistry analysis

Blood samples were collected from patients following an overnight fast of at least 8 h. Blood samples were collected into their corresponding VACUETTE tubes in a randomized drawing order. Tubes coated with a clot activator were used to obtain serum after clotting for 30 min at room temperature followed by centrifugation at 3000 *g* for 5 min. Tubes spray dried with 1.2–2 mg anhydrous EDTA per 1 ml blood were used for determinations of complete blood count (CBC) and glycated haemoglobin (HbA1c). Plasma was obtained immediately after sample collection into heparinized tubes (18 IU of lithium salt of heparin per 1 ml blood), followed by centrifugation at 3000 *g* for 5 min. Serum and plasma samples were transferred into 13 × 75 mm test tubes and stored at − 80 °C for later analysis.

### 1D ^1^H NMR analysis

The protocol of Beckonert et al*.* was followed and adapted for the NMR analysis of plasma samples^39^. An aliquot of 200 μl of fasting plasma was added to 400 μl of 0.9% saline/1% D_2_O buffer. The samples were mixed and then centrifuged at 13,000 rpm for 10 min. A total of 600 μl samples were placed in 5 mm NMR tubes. One-dimensional ^1^H NMR spectra were measured at 500 MHz (Bruker DRX-500 spectrometer) using a standard pre-saturation pulse sequence for water suppression with irradiation at the water frequency during the relaxation delay of 3 s and the pulse sequence mixing time of 100 ms. Spectra were acquired using 64 scans into 64 K points and a spectral width of 7003 Hz, an acquisition time of 4.68 s, and a total pulse recycle time of 7.68 s. Spectra were phase and baseline corrected using TOPSPIN 3.2 (Bruker Analytik, Rheinstetten, Germany) to regions 0.04 ppm wide from δ 10.0 to 0.0. Then, data normalisation and data reduction were performed using AMIX (Bruker Analytik, Rheinstetten, Germany). Alpha-glucose anomeric doublet at δ5.233 was used as a reference to phase the spectra. The reduced and normalised data was scaled using Pareto scaling and a PCA and OPLS-DA models were constructed using SIMCA 15 (Simca v. 15, MKS Umetrics AB, Sweden). Outlier samples that fell outside the Hotelling’s T2 plot were individually analysed and excluded from further multivariate analysis when necessary. Important variables identified from OPLS-DA models as contributing to sample separation, were then matched to chemical shifts in the ^1^H NMR spectra. Identification was carried out first by the interpretation of the NMR spectra, and then by comparing the identified chemical shifts of character peaks, with the chemical shifts available at CHENOMX spectral reference library (Chenomx NMR suite version 8.5, Edmonton, Canada), the Human Metabolome Database (HMDB), and the Biological Magnetic Resonance Data Bank (BMRDB)^40,41^. The information obtained from these resources, the interpretation and comparison with spiked standards, all aided the identification of the potential biomarkers reported in this study.

### Mass spectroscopy analysis (FIA-MS/MS and LC-MS/MS)

A mass spectrometric-based metabolomic approach was carried out at BIOCRATES Life Sciences AG (Eduard-Bodem-Gasse 8, A-6020 Innsbruck, Austria) using the BIOCRATES ABSOLUTE*IDQ* p180 Assay Kit; only 40 samples per group were randomly selected (due to budget limitations) and analysed. The samples cannot be remeasured, and the data doesn’t exist for the excluded 10 samples per group. The assay allows the quantification of 188 metabolites. The kit plates were used for the quantification of amino acids, biogenic amines, acylcarnitines, (lyso-) phosphatidylcholines, sphingomyelins, and hexoses. The fully automated assay was based on phenylisothiocyanate (PITC) derivatization in the presence of internal standards followed by Flow Injection Analysis Tandom Mass Spectrometry (FIA-MS/MS) (acylcarnitines, (lyso-) phosphatidylcholines, sphingomyelins, hexoses) and LC-MS/MS (amino acids, biogenic amines) using a SCIEX 4000 QTRAP (SCIEX, Darmstadt, Germany) or a Waters TQ-S micro (Waters, Vienna) instrument with electrospray ionization (ESI). Metabolites naming and abbreviation was following the description of Römisch-Margl et al.^42^. The experimental metabolomics measurement technique is described in detail by patents EP 1 897 014 B1 and EP 1 875 401 B1^43,44^.

### Statistical analysis

Physical measurements, clinical chemistry parameters (presented as the means and SDs) and area under the curve (AUC) of 1D ^1^H NMR chemical shift regions identified using OPLS-DA models and having VIP values > 1, were compared by the means of a Student’s t-test and values that differ significantly were selected (shown as *P < 0.05, **P < 0.01 and ***P < 0.001). The software package used was SPSS (IBM SPSS statistics 19, Portsmouth, Hampshire, PO6 3AU).

To ensure data quality, different statistical methods were applied on the ABSOLUTE*IDQ* p180 data (MS data) with the aim of identifying differences between the groups. The analysis included data normalization, imputation and transformation followed by univariate statistics with significance testing. The data sets were cleaned to exclude analytes of which concentration values are missing or are below the limit of detection (LOD). The cleaning of the raw data was done by applying a modified 80% rule. Cleaned data was employed for scaling, transformation and the statistical analysis. Missing value imputation was used to replace missing values with a non-zero value while maintaining the overall data structure. The study data was further processed by a log2 transformation to correct for heteroscedasticity, skewedness and improve the interpretability and visualization. Data processing, statistical analysis and data visualization were performed using R (Version 3.2.3). Independent t-tests were performed; a significance level of α = 0.05 was determined, and p-values were calculated. To control the false discovery rate (FDR) during multiple comparisons, an adjusted *P*-value (q value; Benjamini–Hochberg correction) was calculated. Only metabolites with *P*-values lower than 0.05 together with q values lower than 0.05 were reported.

Power was calculated using G* POWER version 3.1.9.4. A post hoc approach was adopted; effect size (Cohen’s d) calculated as difference between two independent means (two groups) where (d = small, ≥ 0.2; medium, ≥ 0.5; large, ≥ 0.8), and the α-level = 0.05 (only 5% chance of producing false positive). Power values are typically accepted at ≥ 80% while values above 95% are preferable and indicate strong power.

Correlation analysis was performed using the cleaned version of MS data for the selected metabolites, and age, sex, BMI, high-density lipoprotein, cholesterol, triglyceride, HbA1c, fasting glucose and fasting insulin levels, were added as covariates. This was performed using GRAPHPAD PRISM (version 7).

## Supplementary information

Supplementary Table.

## Data Availability

The complete datasets generated and/or analysed during the current study are not publicly available due to confidentiality and the terms of the collaboration agreement. However, reasonable requests will be considered by the corresponding authors subject to approval by the responsible authorities at the Ministry of Health and Prevention in the UAE.
