# Supplementary material for: Pre-diagnostic biomarkers of type 2 diabetes identified in the UAE’s obese national population using targeted metabolomics
Source: Sci Rep. 2020 Oct 19;10:17616. doi: 10.1038/s41598-020-73384-7 (PMC7572402; doi:10.1038/s41598-020-73384-7)
Supplement: Supplementary file 1 — Supplementary Table. [file 41598_2020_73384_MOESM1_ESM.docx]

Supplementary information file

**Pre-diagnostic biomarkers of type 2 diabetes identified in the UAE’s obese national population using targeted metabolomics**.

Asma M. Fikri*^13^, Rosemary Smyth^2^, Vijay Kumar^3^, Zainab Abadla^3^, Salahedeen Abusnana^3^ And Michael R. Munday*^2^

^1^Ministry of Health & Prevention, United Arab Emirates

^2^Department of Pharmaceutical and Biological Chemistry, UCL School of Pharmacy, United Kingdom

^3^Rahsid Centre for Diabetes and Research, Ajman, United Arab Emirates

**SUPPLEMENTARY TABLE S1**

| **Number** | **Metabolite Class** | **Metabolite** | **Mean Conc.**  **Control (Obese)** | **Mean Conc.**  **Obese + T2DM** | **p-value** | **q-value**  **(BH adjusted)** |
| --- | --- | --- | --- | --- | --- | --- |
| 1 | Phosphatidylcholines | PC.ae.C36.2 | 9.1177 | 7.251 | 1.9736E-05 | 0.0004 |
| 2 | Phosphatidylcholines | PC.ae.C30.0 | 0.2852 | 0.2228 | 5.3311E-05 | 0.0008 |
| 3 | Phosphatidylcholines | PC.ae.C32.2 | 0.4994 | 0.4118 | 0.0014 | 0.0084 |
| 4 | Phosphatidylcholines | PC.aa.C28.1 | 2.002 | 1.6882 | 0.0017 | 0.0091 |
| 5 | Sphingomyelins | SM.C16.0 | 120.51 | 104.63 | 0.0026 | 0.0130 |
| 6 | Amino acids and Biogenic Amines | Thr | 122.44 | 102.44 | 0.0033 | 0.0164 |
| 7 | Phosphatidylcholines | PC.aa.C34.3 | 11.2078 | 9.4175 | 0.0039 | 0.0187 |
| 8 | Phosphatidylcholines | PC.ae.C32.1 | 2.0205 | 1.7165 | 0.0042 | 0.0196 |
| 9 | Phosphatidylcholines | PC.ae.C34.1 | 6.65 | 5.8338 | 0.0048 | 0.0209 |
| 10 | Phosphatidylcholines | PC.ae.C38.4 | 10.0835 | 8.9802 | 0.0048 | 0.0209 |
| 11 | Amino acids and Biogenic Amines | Ser | 113.4625 | 98.47 | 0.0050 | 0.0210 |
| 12 | Amino acids and Biogenic Amines | Cit | 24.8068 | 20.8397 | 0.0060 | 0.0245 |
| 13 | Sphingomyelins | SM.C18.1 | 14.141 | 11.9947 | 0.0068 | 0.0269 |
| 14 | Amino acids and Biogenic Amines | Tyr | 83.84 | 73.8075 | 0.0072 | 0.0280 |
| 15 | Sphingomyelins | SM.C24.1 | 46.2375 | 40.945 | 0.0108 | 0.0407 |
| 16 | Amino acids and Biogenic Amines | Phe | 64.4175 | 59.0025 | 0.0121 | 0.0440 |
| 17 | Amino acids and Biogenic Amines | Arg | 90.39 | 76.1275 | 0.0123 | 0.0440 |
| 18 | Sphingomyelins | SM.C24.0 | 22.3125 | 20.0825 | 0.0163 | 0.0543 |
| 19 | Phosphatidylcholines | PC.aa.C32.2 | 2.5637 | 2.0393 | 0.0176 | 0.0572 |
| 20 | Sphingomyelins | SM..OH..C24.1 | 0.9732 | 0.8348 | 0.0193 | 0.0613 |
| 21 | Amino acids and Biogenic Amines | Gly | 238.05 | 210.975 | 0.0208 | 0.0647 |
| 22 | Lysophosphatidylcholines | lysoPC.a.C28.1 | 0.3579 | 0.2941 | 0.0226 | 0.0685 |
| 23 | Amino acids and Biogenic Amines | Glu | 99.6275 | 115.735 | 0.0230 | 0.0685 |
| 24 | Phosphatidylcholines | PC.ae.C36.5 | 9.947 | 8.9497 | 0.0337 | 0.0967 |
| 25 | Amino acids and Biogenic Amines | Met | 25.2725 | 22.545 | 0.0338 | 0.0967 |
| 26 | Phosphatidylcholines | PC.ae.C34.0 | 1.1407 | 0.9879 | 0.0376 | 0.1055 |
| 27 | Phosphatidylcholines | PC.ae.C42.4 | 0.705 | 0.6215 | 0.0384 | 0.1056 |
| 28 | Phosphatidylcholines | PC.aa.C38.6 | 56.2925 | 64.4875 | 0.0440 | 0.1186 |
| 29 | Phosphatidylcholines | PC.ae.C38.2 | 1.9417 | 1.723 | 0.0492 | 0.1303 |
| 30 | Acylcarnitines | C2 | 5.9315 | 6.611 | 0.0551 | 0.1424 |
| 31 | Phosphatidylcholines | PC.aa.C36.1 | 34.4675 | 31.415 | 0.0558 | 0.1424 |
| 32 | Acylcarnitines | C18.2 | 0.0533 | 0.0467 | 0.0578 | 0.1446 |
| 33 | Phosphatidylcholines | PC.aa.C40.5 | 7.2577 | 6.4798 | 0.0587 | 0.1446 |
| 34 | Lysophosphatidylcholines | lysoPC.a.C16.0 | 74.045 | 67.795 | 0.0728 | 0.1764 |
| 35 | Sphingomyelins | SM..OH..C22.1 | 14.6685 | 13.417 | 0.0744 | 0.1773 |
| 36 | Amino acids and Biogenic Amines | Asn | 36.48 | 33.94 | 0.0775 | 0.1816 |
| 37 | Lysophosphatidylcholines | lysoPC.a.C17.0 | 1.3068 | 1.155 | 0.0804 | 0.1851 |
| 38 | Phosphatidylcholines | PC.ae.C44.5 | 1.1176 | 1.0081 | 0.0815 | 0.1851 |
| 39 | Lysophosphatidylcholines | lysoPC.a.C28.0 | 11.4165 | 10.2462 | 0.0839 | 0.1875 |
| 40 | Sphingomyelins | SM.C26.0 | 0.1508 | 0.1345 | 0.0935 | 0.2057 |
| 41 | Phosphatidylcholines | PC.aa.C40.6 | 19.4655 | 21.959 | 0.1067 | 0.2282 |
| 42 | Sphingomyelins | SM.C18.0 | 26.195 | 24.01 | 0.1069 | 0.2282 |
| 43 | Amino acids and Biogenic Amines | Asp | 6.0574 | 6.5258 | 0.1116 | 0.2347 |
| 44 | Phosphatidylcholines | PC.ae.C44.4 | 0.2616 | 0.2394 | 0.1151 | 0.2385 |
| 45 | Phosphatidylcholines | PC.aa.C40.3 | 0.4725 | 0.4336 | 0.1238 | 0.2530 |
| 46 | Phosphatidylcholines | PC.aa.C40.4 | 3.419 | 3.1555 | 0.1294 | 0.2586 |
| 47 | Phosphatidylcholines | PC.ae.C40.4 | 2.393 | 2.2215 | 0.1302 | 0.2586 |
| 48 | Phosphatidylcholines | PC.ae.C30.1 | 0.1082 | 0.0894 | 0.1388 | 0.2719 |
| 49 | Phosphatidylcholines | PC.ae.C38.6 | 4.6005 | 4.2432 | 0.1457 | 0.2816 |
| 50 | Acylcarnitines | C14.2 | 0.0312 | 0.035 | 0.1498 | 0.2856 |
| 51 | Phosphatidylcholines | PC.ae.C36.0 | 0.6611 | 0.6163 | 0.1549 | 0.2915 |
| 52 | Acylcarnitines | C10 | 0.1823 | 0.2058 | 0.1595 | 0.2962 |
| 53 | Lysophosphatidylcholines | lysoPC.a.C20.3 | 1.6233 | 1.4433 | 0.1627 | 0.2973 |
| 54 | Amino acids and Biogenic Amines | ADMA | 0.4396 | 0.4159 | 0.1642 | 0.2973 |
| 55 | Phosphatidylcholines | PC.aa.C42.5 | 0.2449 | 0.2321 | 0.1815 | 0.3233 |
| 56 | Acylcarnitines | C18.1 | 0.1255 | 0.1166 | 0.1858 | 0.3233 |
| 57 | Phosphatidylcholines | PC.ae.C44.6 | 0.8839 | 0.8018 | 0.1868 | 0.3233 |
| 58 | Phosphatidylcholines | PC.ae.C42.2 | 0.4613 | 0.4316 | 0.1876 | 0.3233 |
| 59 | Amino acids and Biogenic Amines | Lys | 149.5975 | 157.6325 | 0.2009 | 0.3420 |
| 60 | Phosphatidylcholines | PC.aa.C32.1 | 8.8718 | 11.0523 | 0.2099 | 0.3531 |
| 61 | Acylcarnitines | C0 | 33.44 | 31.865 | 0.2135 | 0.3548 |
| 62 | Acylcarnitines | C18 | 0.0348 | 0.0321 | 0.2167 | 0.3548 |
| 63 | Lysophosphatidylcholines | lysoPC.a.C20.4 | 4.401 | 4.8255 | 0.2183 | 0.3548 |
| 64 | Phosphatidylcholines | PC.aa.C38.5 | 39.075 | 37.05 | 0.2310 | 0.3711 |
| 65 | Amino acids and Biogenic Amines | Kynurenine | 1.9078 | 1.8402 | 0.2355 | 0.3743 |
| 66 | Phosphatidylcholines | PC.aa.C36.5 | 12.779 | 14.782 | 0.2587 | 0.4066 |
| 67 | Phosphatidylcholines | PC.ae.C40.2 | 1.4736 | 1.3828 | 0.2800 | 0.4352 |
| 68 | Phosphatidylcholines | PC.ae.C44.3 | 0.107 | 0.1166 | 0.2852 | 0.4366 |
| 69 | Amino acids and Biogenic Amines | Creatinine | 64.6725 | 61.6525 | 0.2870 | 0.4366 |
| 70 | Amino acids and Biogenic Amines | Taurine | 71.2275 | 77.1875 | 0.3027 | 0.4500 |
| 71 | Phosphatidylcholines | PC.aa.C34.4 | 1.2192 | 1.1345 | 0.3029 | 0.4500 |
| 72 | Acylcarnitines | C3 | 0.2933 | 0.3159 | 0.3052 | 0.4500 |
| 73 | Phosphatidylcholines | PC.aa.C32.0 | 10.4627 | 10.0883 | 0.3114 | 0.4544 |
| 74 | Sphingomyelins | SM.C26.1 | 0.2799 | 0.2651 | 0.3190 | 0.4608 |
| 75 | Phosphatidylcholines | PC.ae.C38.1 | 0.7612 | 0.7301 | 0.3461 | 0.4949 |
| 76 | Phosphatidylcholines | PC.aa.C30.0 | 2.9373 | 2.7967 | 0.4067 | 0.5672 |
| 77 | Lysophosphatidylcholines | lysoPC.a.C26.0 | 0.2444 | 0.2105 | 0.4069 | 0.5672 |
| 78 | Acylcarnitines | C5 | 0.1166 | 0.1107 | 0.4122 | 0.5672 |
| 79 | Phosphatidylcholines | PC.aa.C36.0 | 1.2031 | 1.2759 | 0.4125 | 0.5672 |
| 80 | Phosphatidylcholines | PC.ae.C38.0 | 1.5971 | 1.7042 | 0.4493 | 0.5951 |
| 81 | Phosphatidylcholines | PC.aa.C36.6 | 0.5511 | 0.5972 | 0.4501 | 0.5951 |
| 82 | Amino acids and Biogenic Amines | Leu | 137.44 | 142.67 | 0.4526 | 0.5951 |
| 83 | Phosphatidylcholines | PC.ae.C36.1 | 5.5145 | 5.3148 | 0.4534 | 0.5951 |
| 84 | Amino acids and Biogenic Amines | Met.SO | 1.777 | 1.8635 | 0.4546 | 0.5951 |
| 85 | Acylcarnitines | C4 | 0.179 | 0.1831 | 0.4577 | 0.5951 |
| 86 | Amino acids and Biogenic Amines | Ile | 90.9025 | 93.4675 | 0.4620 | 0.5953 |
| 87 | Amino acids and Biogenic Amines | Sarcosine | 1.1273 | 1.1936 | 0.4898 | 0.6254 |
| 88 | Phosphatidylcholines | PC.aa.C42.6 | 0.2698 | 0.2629 | 0.5014 | 0.6311 |
| 89 | Amino acids and Biogenic Amines | Serotonin | 0.1268 | 0.1164 | 0.5031 | 0.6311 |
| 90 | Amino acids and Biogenic Amines | Putrescine | 0.0892 | 0.0949 | 0.5085 | 0.6324 |
| 91 | Amino acids and Biogenic Amines | Val | 237.95 | 244.275 | 0.5155 | 0.6355 |
| 92 | Lysophosphatidylcholines | lysoPC.a.C16.1 | 1.975 | 1.9433 | 0.5537 | 0.6767 |
| 93 | Phosphatidylcholines | PC.aa.C36.4 | 155.5825 | 160.73 | 0.5643 | 0.6838 |
| 94 | Phosphatidylcholines | PC.aa.C42.1 | 0.2042 | 0.2117 | 0.5760 | 0.6922 |
| 95 | Phosphatidylcholines | PC.ae.C42.5 | 1.663 | 1.639 | 0.5897 | 0.7027 |
| 96 | Phosphatidylcholines | PC.aa.C38.4 | 81.615 | 80.87 | 0.6274 | 0.7415 |
| 97 | Lysophosphatidylcholines | lysoPC.a.C26.1 | 0.1678 | 0.1561 | 0.6513 | 0.7634 |
| 98 | Phosphatidylcholines | PC.aa.C38.0 | 1.9522 | 2.0092 | 0.6683 | 0.7770 |
| 99 | Phosphatidylcholines | PC.ae.C40.3 | 1.7462 | 1.8027 | 0.6814 | 0.7858 |
| 100 | Amino acids and Biogenic Amines | alpha.AAA | 1.0814 | 1.1049 | 0.6887 | 0.7879 |
| 101 | Phosphatidylcholines | PC.aa.C42.4 | 0.193 | 0.202 | 0.7002 | 0.7946 |
| 102 | Phosphatidylcholines | PC.ae.C42.1 | 0.3776 | 0.3657 | 0.7183 | 0.8088 |
| 103 | Amino acids and Biogenic Amines | Pro | 223.65 | 217.47 | 0.7263 | 0.8114 |
| 104 | Phosphatidylcholines | PC.aa.C38.1 | 0.6039 | 0.6098 | 0.7404 | 0.8158 |
| 105 | Phosphatidylcholines | PC.aa.C42.2 | 0.1794 | 0.185 | 0.7416 | 0.8158 |
| 106 | Amino acids and Biogenic Amines | t4.OH.Pro | 8.3368 | 8.8123 | 0.7644 | 0.8304 |
| 107 | Acylcarnitines | C14.1 | 0.0949 | 0.0974 | 0.7665 | 0.8304 |
| 108 | Phosphatidylcholines | PC.ae.C40.5 | 3.1298 | 3.222 | 0.8231 | 0.8850 |
| 109 | Acylcarnitines | C16 | 0.1047 | 0.1033 | 0.8349 | 0.8861 |
| 110 | Phosphatidylcholines | PC.aa.C34.1 | 140.6 | 141.2625 | 0.8365 | 0.8861 |
| 111 | Amino acids and Biogenic Amines | Spermidine | 0.1811 | 0.1804 | 0.8623 | 0.8991 |
| 112 | Phosphatidylcholines | PC.ae.C40.1 | 1.1345 | 1.1517 | 0.8704 | 0.8991 |
| 113 | Phosphatidylcholines | PC.aa.C42.0 | 0.3669 | 0.3717 | 0.8707 | 0.8991 |
| 114 | Phosphatidylcholines | PC.ae.C38.3 | 4.4058 | 4.3333 | 0.8784 | 0.8991 |
| 115 | Phosphatidylcholines | PC.aa.C40.2 | 0.3295 | 0.3332 | 0.8803 | 0.8991 |
| 116 | Phosphatidylcholines | PC.ae.C40.6 | 3.0795 | 3.0795 | 0.9283 | 0.9415 |
| 117 | Phosphatidylcholines | PC.ae.C42.3 | 0.6272 | 0.6347 | 0.9672 | 0.9740 |
| 118 | Amino acids and Biogenic Amines | SDMA | 0.3918 | 0.3946 | 0.9948 | 0.9948 |
